# Designing New Material Based on Functionalized Multi-Walled Carbon Nanotubes and Cu(OH)_2_–Cu_2_O/Polypyrrole Catalyst for Ethanol Oxidation in Alkaline Medium

**DOI:** 10.3389/fchem.2021.805654

**Published:** 2022-02-04

**Authors:** Anas El Attar, Sanaa Chemchoub, Mamadou Diallo Kalan, Larbi Oularbi, Mama El Rhazi

**Affiliations:** Laboratory of Materials, Membranes, and Environment, Faculty of Science and Technology, University Hassan II of Casablanca, Mohammedia, Morocco

**Keywords:** functionalized multi-walled carbon nanotube, carbon paste electrode, copper hydroxide–copper oxide, polypyrrole, ethanol oxidation

## Abstract

In this work, copper(II) hydroxide (Cu(OH)_2_) and copper oxide (Cu_2_O) nanostructures are deposited on functionalized multi-walled carbon nanotubes/polypyrrole to report an efficient electrocatalyst for ethanol oxidation in alkaline medium. In the first step, the deposition of functionalized multi-walled nanotubes of carbon (F-MWCNTs) on the electrode surface was carried out using drop casting mode followed by the electrodeposition of polypyrrole (PPy) and copper nanoparticles (Cu-Nps) using galvanostatic mode. Scanning electron microscopy (SEM) and X-ray diffraction (XRD) were performed in order to study the morphology and the structure of the elaborated catalysts. Electrochemical characterization conducted by cyclic voltammetry (CV) and electrochemical impedance spectroscopy (EIS) revealed that the introduction of functionalized multi-walled carbon nanotubes enhances the electric properties of the nanocomposites and offers a large active surface area. The prepared electrocatalyst was then tested in a solution of 0.1 M NaOH containing 0.2 M of ethanol showing high performance (7 mA cm^−2^ at 0.85 V vs Ag/AgCl) and good stability (over 1800 s) toward ethanol oxidation.

## Introduction

Developing a new and cost-effective source of energy is key to keeping up with the growing global demand for energy (El-Houari et al., 2020; Rahman and Velayutham, 2020; Ivanovski et al., 2021). In response to the rising energy demand, different strategies have been adopted. Among them, fuel cells have been considered a promising candidate. Indeed, direct ethanol fuel cells (DEFCs) appear to be a good alternative and offer several benefits compared to the other types of fuel cells (Guo et al., 2018). Their excellent volumetric energy density (8 kWh kg^−1^) (Eisa et al., 2020), ease of transportation and storage (Fontes et al., 2019; Souza et al., 2020), low toxicity (Mondal et al., 2019), and large availability of ethanol have made them extensively studied as potential tools for portable mobile devices and in automobile fields (Silva et al., 2019; Xaba et al., 2019). However, two crucial issues hinder the practical uses of DEFCs: i) the sluggish kinetic of ethanol oxidation reaction (EOR) and the poisoning of catalyst surface caused by the formation of intermediate species during ethanol oxidation (Cermenek et al., 2019; Choudhary and Pramanik, 2020a; Choudhary and Pramanik, 2020b) and ii) the exorbitant price of catalysts (Liang et al., 2021). The design of new materials with high performance and low cost still remains a serious challenge for researchers. In this context, electrocatalysts based on carbon nanomaterials/conductive polymers as a support for metal nanoparticles have been studied (Fard et al., 2017; Ghosh et al., 2017; Boulaghi et al., 2018; Mozafari and Parsa, 2020a), which were synthetized as an appropriate support for ethanol oxidation in acid medium based on polyaniline and carbon nanotube (PANI/CNT) for platinum (Pt) particles. It has been proved that the combination of PANI and CNT increases the active surface area of the catalyst and ensures a good dispersion of platinum nanoparticles onto the polymer (De et al., 2017). In the same way, a facile synthesis of polyaniline–multi-walled carbon nanotubes–tin oxide on the titanium (Ti) mesh substrate was reported to provide a support for the palladium (Pd) catalyst (Mozafari and Parsa, 2020a). The high performance and good stability of the catalyst were directly related to the presence of MWCNT and SnO_2_ inside the polymer. Recently, the polypyrrole/MWCNT nanocomposite–modified glassy carbon electrode was also used as a support for PdCo porous nanostructures (PNSs) leading to a better catalytic activity and high tolerance to poisoning of the surface by the intermediate species (Fard et al., 2017). It seems that the presence of carbon nanomaterials as a support for conductive polymers offers a high active surface area, while the presence of an amino-group in the backbone of conducting polymers ensures a good deposition of metal particles and facilitates accessibility to electrocatalytic sites of ethanol (Chemchoub et al., 2020; El Attar et al., 2020). However, very few studies have been devoted to a non-noble metal combined with carbon nanostructures and polymers. In our previous study, we studied the effect of the activation of the surface of Cu_2_O-NDs/PPy/CPE and Cu_2_O/PPy/CPE electrocatalysts on the performance of EOR. It has been demonstrated that the regeneration of copper in acidic medium plays an important role in the morphology of the material (copper oxide) varying from the octahedral particles to the dendritic one. This change in the morphology leads to an increase of the performance of the catalyst in terms of stability and durability toward ethanol oxidation (El Attar et al., 2020; El Attar et al., 2021). In this study, our goal is to explore the effect of F-MWCNTs on deposition of copper hydroxide–copper oxide nanoparticles. To the best of our knowledge, no studies have been devoted to the effect of deposition of copper hydroxide–copper oxide nanodendrites on PPy/F-MWCNTs for ethanol oxidation. In this context, we report for the first time a facile synthesis of the Cu(OH)_2_–Cu_2_O/PPy/F-MWCNT–modified carbon paste electrode (CPE) for ethanol oxidation. Two steps are required for the elaboration of the catalyst: The first step consists of the deposition of functionalized multi-walled carbon nanotubes on the surface of carbon paste electrode followed by polymerization of pyrrole by galvanostatic mode. Copper hydroxide–copper oxide is then electrodeposited on PPy/F-MWCNTs in the second step using galvanostatic mode followed by polarization of the electrode in alkaline solution. After carefully examining the morphology and the electrochemical properties of the electrocatalyst, nanocomposites are then applied for ethanol oxidation. The results of experimental studies indicated that Cu(OH)_2_–Cu_2_O dispersed onto the PPy/F-MWCNT/CPE catalyst shows excellent catalytic activity, high electrical conductivity, and long stability toward ethanol oxidation.

## Materials and Methods

### Chemicals

Pyrrole (99%) was obtained from ACROS Organics and was purified by distillation prior to usage. Lithium perchlorate (LiClO_4_; purum p.a. 98%), potassium ferri/ferrocyanide (K_3_Fe(CN)_6_/K_4_Fe(CN)_6_·3H_2_O; ACS reagent ≥99%), MWCNTs with an outside diameter of 6–13 nm and a length of 2.5–20 μm, graphite powder with the particle size less than 20 μm, paraffin oil, graphite powder with the size of particles <20 μm, copper(II) chloride dihydrate (CuCl_2_·2H_2_O), and sodium sulfate (Na_2_SO_4_) were obtained from SIGMA-ALDRICH. Nitric acid (HNO_3_; 68%) was procured from AnalaR NORMAPUR and sulfuric acid (H_2_SO_4_; 98%) from Fluka. Potassium chloride (KCl), sodium hydroxide (NaOH) (98%), and ethanol (99.98%) were obtained from VWR PROLABO CHEMICALS.

### Synthesis of Cu(OH)_2_–Cu_2_O Modified PPy/CPE and PPy/F-MWCNTs/CPE

#### Functionalization of the MWCNTs

An appropriate amount of MWCNTs was dispersed in a mixture of concentrated H_2_SO_4_ and HNO_3_ at a volume ratio of 1:3 under ultrasonic agitation for few minutes and then refluxed at 80 °C under magnetic stirring for 4 h. The MWCNTs were then filtered on a Millipore polycarbonate membrane (Ø 0.22 μm) and washed with bi-distilled water until the filtrate reaches a neutral pH value. Finally, the functionalized MWCNTs were dried under vacuum at 50°C for 5 h.

#### F-MWCNTs Modified CPE

A suspension of F-MWCNTs with a concentration of 1 mg ml^−1^ was prepared by dispersing the functionalized MWCNT in bi-distilled water under ultrasonic vibration. Then, a volume of 10 μl of the dispersed F-MWCNTs was dropped on the CPE surface and dried at 40 °C for 20 min. The modified electrode was named “F-MWCNTs/CPE.”

#### PPy Modified Bare CPE and F-MWCNTs/CPE

Electrochemical deposition of PPy on bare CPE and F-MWCNTs/CPE was performed in an aqueous solution containing 0.1 M pyrrole and 0.5 M LiClO_4_ using galvanostatic mode at a current density of 0.2 mA cm^−2^ for 40 s (Oularbi et al., 2019). The modified electrodes were named “PPy/CPE” and “PPy/F-MWCNTs/CPE,” respectively.

#### Cu(OH)_2_–Cu_2_O Modified PPy/CPE and PPy/F-MWCNTs/CPE

Electrochemical deposition of Cu on PPy/CPE and PPy/F-MWCNTs/CPE was performed in an aqueous solution containing 0.1 M CuCl_2_ and 0.1 M Na_2_SO_4_ using galvanostatic mode at a constant current of −225 µA for 60 s. The electrodes were then polarized in a solution of 0.1 M NaOH by applying five cyclic sweeps between −0.2 and 1 V in order to form copper oxide. The modified electrodes were named “Cu(OH)_2_–Cu_2_O/PPy/CPE” and “Cu(OH)_2_–Cu_2_O/PPy/F-MWCNTs/CPE,” respectively.

#### Morphological, Structural, and Elemental Characterization

The morphological properties of the copper oxide catalysts were characterized using a scanning electron microscope (FEI FEG 450) coupled to an EDX spectrum (BRUKER XFlash 6/30). An IRAffinity-1S SHIMADZU Fourier transform infrared (FTIR) spectrophotometer was equipped with a Golden Gate single reflection attenuated total reflectance (ATR) accessory. FTIR spectra were recorded in the range of 500–4000 cm^−1^ at a resolution of 16 cm^−1^ and were used to determine the structural properties of the catalysts. A PANalytical X-ray diffractometer (XRD) X'PERT PRO MPD, with Cu Kα = 1.540598 Å and operating at 45 kV and 30 mA, was used on the prepared electrode to determine the crystalline phase of Cu(OH)_2_–Cu_2_O supported on PPy/F-MWCNTs/CPE.

### Electrochemical Measurements

All electrochemical measurements including cyclic voltammetry (CV), chronoamperometry, and electrochemical impedance spectroscopy (EIS) were performed using PalmSens4 controlled with PSTrace software version 5.8. The assembly used comprises an electrochemical cell, which contains three electrodes: the modified carbon paste electrode (CPE) as a working electrode, Ag/AgCl as a reference electrode, and a platinum electrode as an auxiliary electrode.

## Results and Discussions

### Physicochemical Characterization of F-MWCNTs

The presence of functional groups of MWCNTs was investigated by Fourier transform infrared (FTIR) spectrum before and after treatment with acid (Supplementary Figure S1A).

As can be seen, the functionalized MWCNTs show new peaks compared to the untreated one. The bands observed at 3453, 1747, and 1098 cm^−1^ correspond to carboxylic groups (-COOH) and are attributed, respectively, to the elongation vibration of hydroxyl (-OH), carbonyl (C=O), and epoxy (C-O) groups. The peaks at 2925 and 2865 cm^−1^ are attributed to the symmetrical and asymmetrical C-H band vibrations produced at the defect sites of the MWCNT surface during acid treatment. The peak at 1648 cm^−1^ is attributed to the elongation vibration of the C=C double band linked to the carbon nanotube structure (Choudhary and Pramanik, 2020a). The peak observed at 2366 cm^−1^ corresponds to the elongation vibration of the strongly hydrogenated -OH bond of the carboxylic groups (─COOH) as mentioned by Oularbi et al. during the preparation of nanocomposites consisting of bismuth particles, polypyrrole, and multi-walled carbon nanotubes (Oularbi et al., 2019). Our results are in good agreement with those of the previous works in the literature confirming that the treated MWCNTs have been successfully oxidized with new functional groups on their surface (De et al., 2017; Adewoye et al., 2021; Qazi et al., 2021).

The morphological structure of MWCNTs after functionalization was examined using scanning electron microscopy (SEM) characterization. Supplementary Figure S1B shows a porous and fibrous nanostructure without impurities which are removed after the acid treatment.

### Electrodes Preparation and Characterization

**FIGURE 1:**
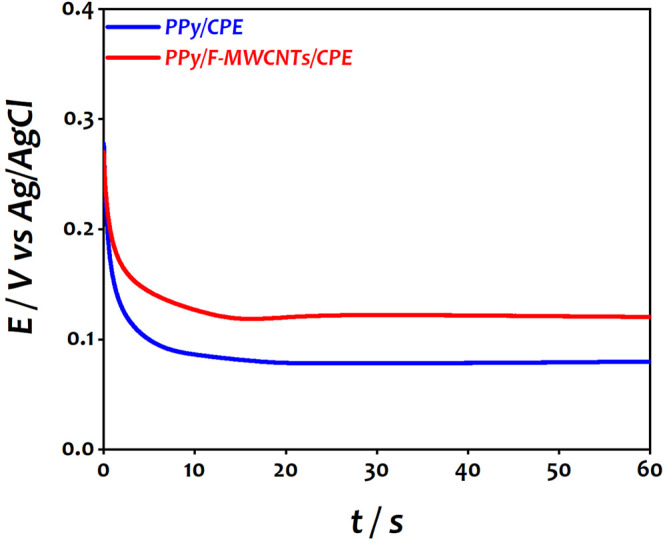
Chronopotentiometric curve of PPy on CPE and F-MWCNTs/CPE during the electropolymerization process.

After dispersion of F-MWCNTs on the electrode surface, the first step consists of the electropolymerization of the PPy film on CPE and F-MWCNTs/CPE using galvanostatic mode in 0.5 M LiClO_4_ in the presence of 0.1 M pyrrole solution. Figure 1 shows the chronopotentiometric curve recorded during the polymerization of polypyrrole on both electrodes.

As can be seen, the potential decreased gradually during the few first seconds and then reached a constant potential of 650 mV vs Ag/AgCl for CPE and 590 mV vs Ag/AgCl for F-MWCNTs/CPE, which corresponds to the growth of the polypyrrole film on different electrodes. The difference of the potential can be attributed to the presence of charge carriers on the F-MWCNT sheets, indicating an interaction between the F-MWCNT sheets and the PPy chain during electropolymerization of PPy. Moreover, the functional group of MWCNTs enhanced the charge transfer during polymerization leading to reduction of the deposition potential. This result is similar to the results reported in the literature during the polymerization of PPy on carbon nanofibers (CNFs) and carbon nanotubes (CNTs) (Oularbi et al., 2017; El Rhazi et al., 2018; Oularbi, 2018; Oularbi et al., 2019). After the polymerization of PPy on both surfaces, the electrochemical behavior of the modified electrodes was investigated in aqueous solution containing 0.5 M LiClO_4_ using cyclic voltammetry (CV) showing a higher current and a well-defined redox behavior on PPy/F-MWCNTs/CPE with a decrease of the value of peak-to-peak separation (***∆E***_***p***_) of about 110 mV compared to that of PPy/CPE (Supplementary Figure S2). These results confirm the role of F-MWCNTs of promoting the electron transfer of the PPy film by providing a high active surface area and an easier electron transfer as reported by other authors (Rizi et al., 2021; Zeng et al., 2021).

**FIGURE 2:**
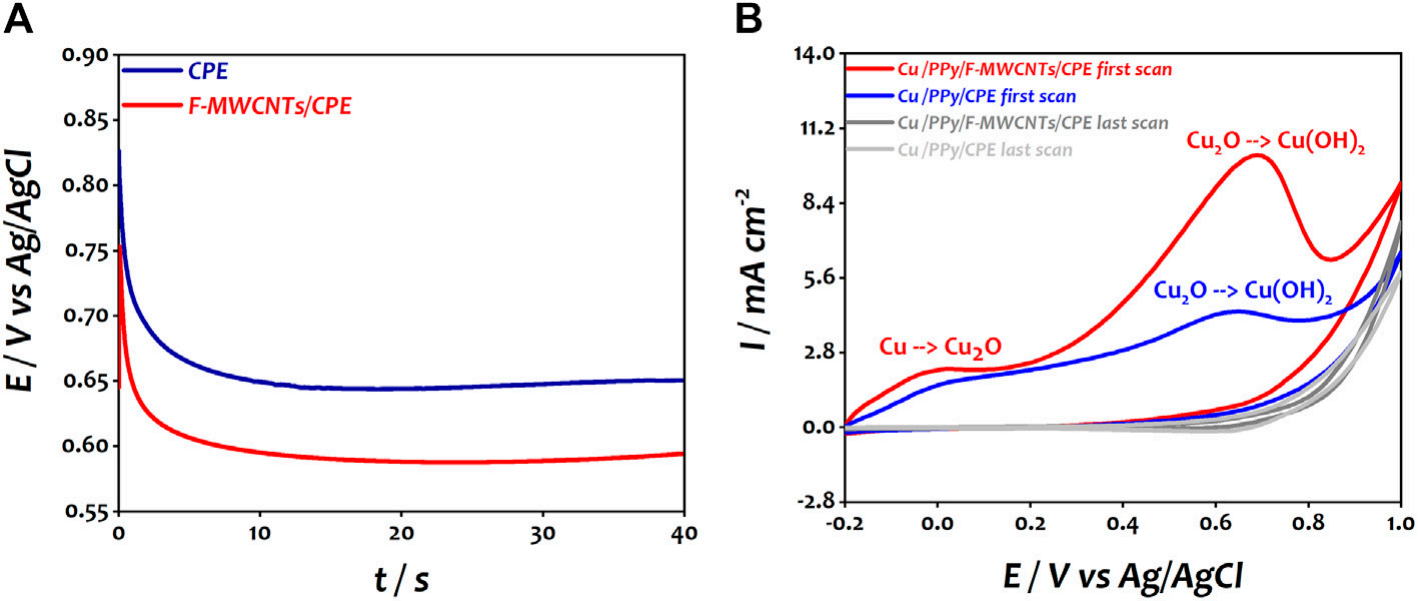
**(A)** Chronopotentiometric curve of CuCl_2_ on PPy/CPE and PPy/F-MWCNTs/CPE during the electrodeposition process. **(B)** Cyclic voltammetry of Cu/PPy/CPE and Cu/PPy/F-MWCNTs/CPE in 0.1 M NaOH at a scan rate of 50 mVs^−1^.

In the second step, the copper nanoparticles were deposited on PPy/CPE and PPy/F-MWCNTs/CPE by applying a constant current of −225 µA for 60 s. As shown in Figure 2A, the deposition process of copper can be divided into two steps according to the potential variation characteristics. In the first step, the potential decreases rapidly on both electrodes which can possibly be due to the double layer charging and the initial nucleation of copper particles (Qiao and West, 2014; Yang et al., 2017). However, we can note that the initial potential for PPy/CPE is around 270 mV, while this value is around 274 mV on PPy/F-MWCNTs/CPE. Then, in the second stage, from 20 s onward, the potential remains practically stable at 80 and 120 mV for PPy/CPE and PPy/F-MWCNTs/CPE, respectively, indicating the deposition of Cu(0) on both electrodes. It should be noted that the nucleation sites of copper covered rapidly the surface of PPy/F-MWCNTs/CPE compared to PPy/CPE, indicating the high conductivity of the PPy/F-MWCNT composite. The increase of potential value at the end of electrodeposition of copper nanoparticles on PPy/F-MWCNTs/CPE confirms that the deposition of copper nanoparticles is easier on the electrode modified by F-MWCNTs owing to their good conductivity (Pérez-Fernández et al., 2017).

Right after that and in order to allow these particles to be oxidized into copper hydroxide and copper oxide, cyclic voltammetry was performed in 0.1 M NaOH solution under the potential range of −0.2 to 1 V vs Ag/AgCl at a scan rate of 50 mV s^−1^ for five cycles for both the electrodes. Figure 2B shows the first and five voltammograms obtained at Cu/PPy/CPE and Cu/PPy/F-MWCNTs/CPE.

The first voltammogram of polarization at both the electrodes shows two anodic peaks in agreement with our previous work (El Attar et al., 2020). The first anodic peak (30 mV vs Ag/AgCl at Cu/PPy/CPE and 0 mV vs Ag/AgCl at Cu/PPy/F-MWCNTs/CPE) is attributed to the oxidation of Cu(0) to Cu(I), which corresponds to Cu_2_O form (Eq. 1) (Stępniowski et al., 2019). The second peak (650 mV vs Ag/AgCl at Cu/PPy/CPE and 690 mV vs Ag/AgCl at Cu/PPy/F-MWCNTs/CPE) corresponds to the oxidation of Cu_2_O to Cu(OH)_2_ as mentioned in the following equation (Moharam et al., 2016):

$$2Cu+2OH^{-}\to Cu_{2}O\;+\;H_{2}O\;+\;2e^{-}$$ (1)

$$Cu_{2}O+H_{2}O+\;2\;OH^{-}\to 2Cu{\left(OH\right)}_{2}+2e^{-}$$ (2)

It should be noted that the anodic peaks at Cu/PPy/F-MWCNTs/CPE are well defined compared to those at Cu/PPy/CPE which can be attributed to the good deposition of copper particles on the modified electrode by the polymer and F-MWCNTs. However, for both electrodes, the current of the anodic peaks decreased progressively for the subsequent cycles, indicating the growth of a passive and stable film formed by copper hydroxide and copper oxide on the surface of the electrodes (Marathey et al., 2019; Stępniowski et al., 2019).

**FIGURE 3:**
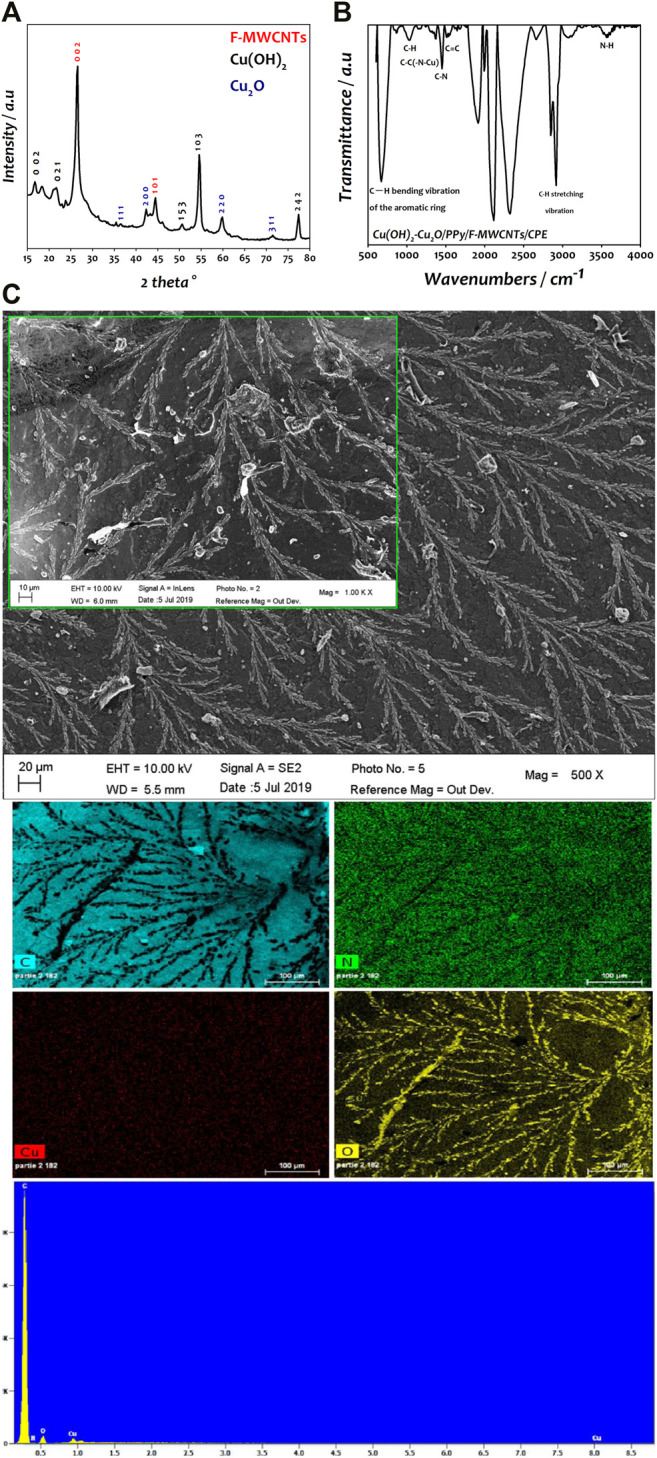
**(A)** XRD analysis of Cu(OH)_2_–Cu_2_O/PPy/F-MWCNTs/CPE. **(B)** FTIR analysis of Cu(OH)_2_–Cu_2_O/PPy/F-MWCNTs/CPE. **(C)** SEM image of Cu(OH)_2_–Cu_2_O/PPy/F-MWCNTs/CPE and EDS and EDX elemental mapping of Cu(OH)_2_–Cu_2_O/PPy/F-MWCNTs/CPE.

The X-ray diffraction (XRD) was used to characterize the crystalline phase of copper deposited on PPy/F-MWCNT/CPE support and is reported in Figure 3A. The spectrum presents well-defined peaks located at 26.57° (002 plane) and 44.42° (101 plane) attributed to the hexagonal structure of F-MWCNTs. The XRD pattern also revealed the presence of two crystalline phases of copper. The diffraction peaks at 16.8° (002 plane), 22.6° (021 plane), 50.7° (153 plane), 54.53° (103 plane), and 77.2° (311 plane) corresponded to the orthorhombic Cu(OH)_2_ (JCPDS 01-072-0140) (Shahrokhian et al., 2019). The peaks at 36.4° (111 plane), 42.4° (200 plane), 59.8° (220 plane), and 71.4° (311 plane) were indexed to the cubic Cu_2_O (JCPDS 01-077-0199) (Aguilar and Rosas, 2019). These results are similar to those obtained by Halim et al. (2021) when using polyphenylenediamine/carbon nanofiber (PpPD/CNF) as a support of Cu_2_O–Cu(OH)_2_ nanoparticles for methanol oxidation in alkaline medium (Halim et al., 2021)**.**

The modified electrodes were investigated by FTIR spectroscopy (Figure 3B), which show different characteristic bands. The peaks at 666, 1018, and 2908 cm^−1^ corresponded to C-H vibration bands of the aromatic ring and C-H stretching vibration of PPy. The peaks observed at 1450 cm^−1^, 3422 cm^−1^, and 1504 cm^−1^ were attributed to C-N, N-H, and C=C, respectively, confirming the effective polymerization of the polypyrrole film on the electrode (Brachetti-Sibaja et al., 2021; Rizi et al., 2021). The peak at 1365 cm^−1^ was attributed to the C-C(-N-Cu) stretching vibration indicating the deposition of copper hydroxide/copper oxide on the surface of the PPy film (Guo et al., 2018; Peshoria and Narula, 2018). Based on the above analysis, we can conclude that Cu(OH)_2_–Cu_2_O/PPy/F-MWCNTs have been successfully synthesized on the modified electrode.

The morphological shape of the Cu(OH)_2_–Cu_2_O nanoparticles deposited on the surface of PPy/F-MWCNTs/CPE was characterized using scanning electron microscopy (SEM) (Figure 3C). As can be seen, Cu(OH)_2_–Cu_2_O was homogeneously deposited on PPy/F-MWCNTs with a three-dimensional nanodendritic structure constituted by multiple symmetrical branches attached to a pronounced central backbone. This kind of morphology is due to the presence of Cl^−^ ion present in copper solution as reported in the literature (Zhuo et al., 2017; El Attar et al., 2021; Halim et al., 2021).

The EDS and EDX mapping images of Cu(OH)_2_–Cu_2_O/PPy/F-MWCNTs/CPE (Figure 3C) show that the carbon element is well proportioned in the electrode surface, due to the presence of F-MWCNTs and the graphite flakes of the bare CPE. The nitrogen elements originating from PPy are well dispersed on the surface and certify the assembly of PPy thin film on the F-MWCNTs. Moreover, a homogeneous dispersion of copper element and the presence of oxygen confirm the formation of Cu(OH)_2_–Cu_2_O on the PPy/F-MWCNT/CPE surface, generating more active sites with more catalytic properties.

**FIGURE 4:**
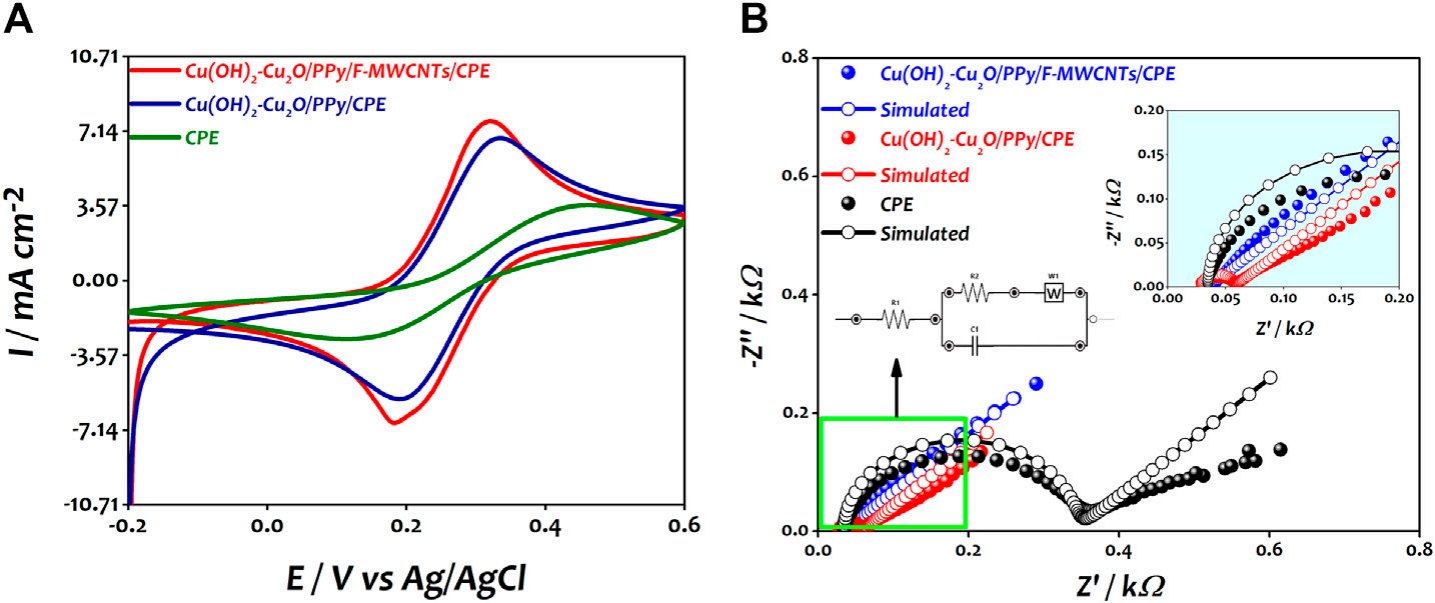
**(A)** Typical cyclic voltammograms of CPE, Cu(OH)_2_–Cu_2_O/PPy/CPE, and Cu(OH)_2_–Cu_2_O/PPy/F-MWCNTs/CPE in 10 mM [Fe(CN)_6_]^3/4-^ in 0.5 M KCl at a scan rate of 100 mV s^−1^. **(B)** Nyquist plots of CPE, Cu(OH)_2_–Cu_2_O/PPy/CPE, and Cu(OH)_2_–Cu_2_O/PPy/F-MWCNTs/CPE obtained in 10 mM [Fe(CN)_6_]^3/4-^ in 0.5 M KCl at 0.25 V.

An electrochemical characterization was performed to evaluate the effect of introducing F-MWCNTs on the electrochemical properties of the modified Cu(OH)_2_–Cu_2_O/PPy/CPE. Therefore, the CVs of bare CPE, Cu(OH)_2_–Cu_2_O/PPy/CPE and Cu(OH)_2_–Cu_2_O/PPy/F-MWCNTs/CPE were recorded in 0.5 M KCl solution containing 10 mM of [Fe(CN)_6_]^3/4-^ as shown in Figure 4A.

**FIGURE 5:**
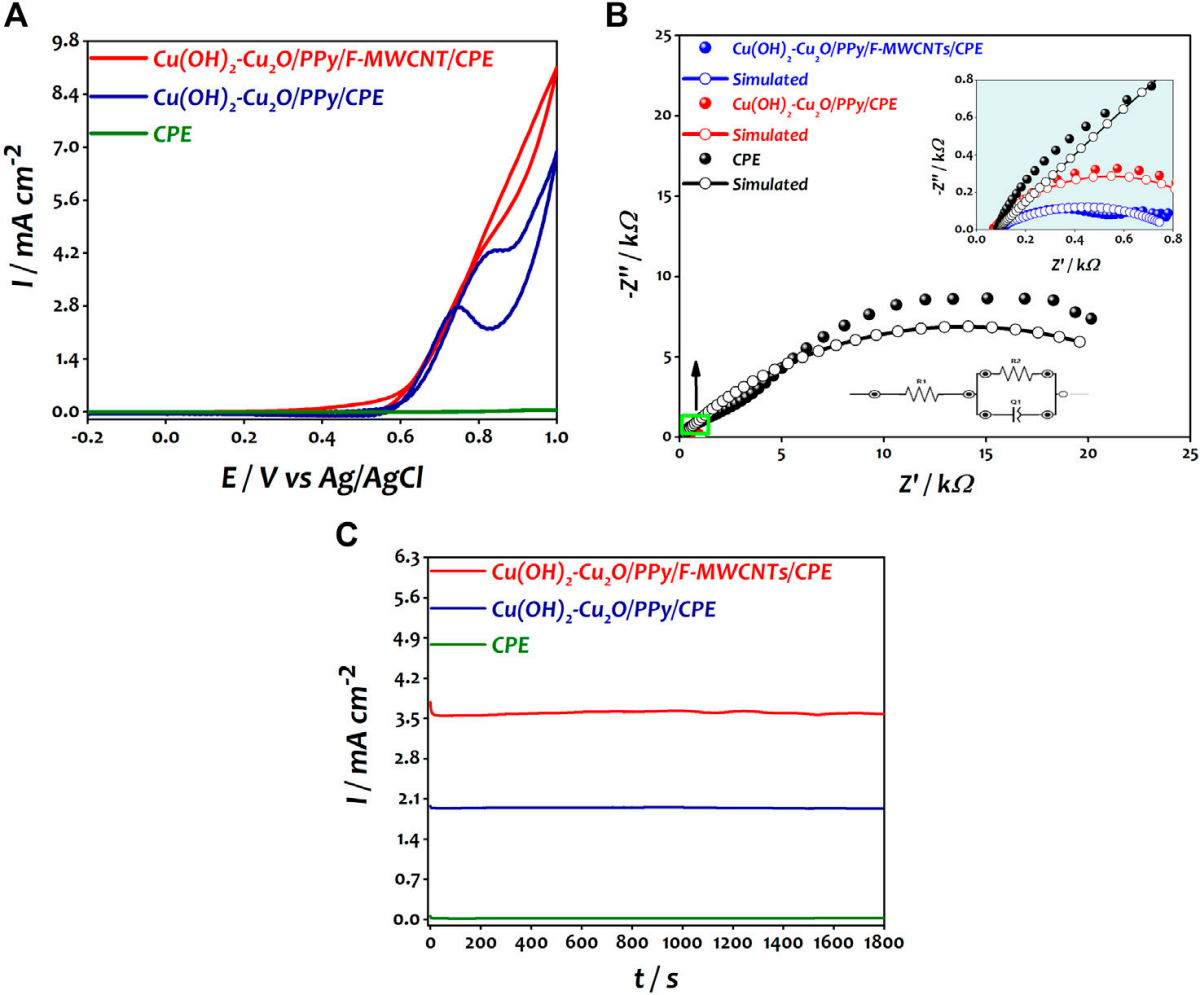
**(A)** Catalytic response of CPE, Cu(OH)_2_–Cu_2_O/PPy/CPE, and Cu(OH)_2_–Cu_2_O/PPy/F-MWCNTs/CPE obtained in 0.2 M ethanol in 0.1 M NaOH. **(B)** Nyquist plots of CPE, Cu(OH)_2_–Cu_2_O/PPy/CPE, and Cu(OH)_2_–Cu_2_O/PPy/F-MWCNTs/CPE obtained in 0.2 M ethanol in 0.1 M NaOH at 0.6 V. **(C)** Chronoamperometric curves of CPE, Cu(OH)_2_–Cu_2_O/PPy/CPE, and Cu(OH)_2_–Cu_2_O/PPy/F-MWCNTs/CPE obtained in 0.2 M ethanol in 0.1 M NaOH at 0.7 V.

As can be seen, the CV curves of the three electrodes show a pair of redox peaks corresponding to the reversible reaction of [Fe(CN)_6_]^3/4-^. The bare CPE has a small redox peak and a high peak-to-peak potential separation (∆Ep) of about 340 mV due to the relatively small surface area and low electron transfer (Salih et al., 2017a; Salih et al., 2017b; Salih et al., 2018; Oularbi et al., 2020). For the Cu(OH)_2_–Cu_2_O/PPy/CPE, the current densities of the anodic and cathodic peaks increased and the ∆Ep value was reduced to 160 mV compared to those of the bare CPE, indicating that the combination of the PPy film and copper oxide nanoparticles improved the conductivity of the electrode surface in agreement with our previous work (El Attar et al., 2020). In the case of Cu(OH)_2_–Cu_2_O/PPy/F-MWCNTs/CPE, the current density increased significantly by about 35.7% compared to that of bare CPE and 12.08% compared to that of Cu(OH)_2_–Cu_2_O/PPy/CPE and the ∆Ep value was reduced to 120 mV. This result could be explained by the positive synergistic effect between F-MWCNTs and PPy leading to the enhancement of the surface area and improvement of the electronic transfer at the electrode surface as suggested by many authors (Fard et al., 2017; Kathiresan et al., 2021). The electrochemical parameters of the different electrodes recorded from the CV curves (Figure 5A) are reported in Table 1.

**TABLE 1:** Characterization of anodic and cathodic peaks of modified electrodes.

Electrode	*I*_pa_ (mA cm^−2^)	*I*_pc_ (mA cm^−2^)	*E*_pa_ (mV)	*E*_pc_ (mV)	*∆E*_*p*_ (mV)
Bare CPE	3.6	−2.8	460	120	340
Cu(OH)_2_–Cu_2_O/PPy/CPE	6.7	−5.62	340	180	160
Cu(OH)_2_–Cu_2_O/PPy/F-MWCNTs/CPE	7.62	−6.80	320	190	130

Since the enhancement of current is directly related to the electrochemically active surface area (EASA) of the electrode, we calculated it by using the Randles–Sevcik equation (Eq. 3), for different electrodes (Abbas et al., 2018):

$$i_{pa}=2.69\times 10^{5}n^{3/2}AC\;D^{1/2}V^{1/2},$$ (3)

where i_pa_ is the anodic current (A), n is the number of electrons transferred (n = 1), A is the electroactive surface area (cm^2^), C is the solution concentration (mol/cm^3^), *V* is the potential scan rate (V s^−1^), and D is the diffusion coefficient (cm^2^/s). The calculated electroactive surface area was 0.086, 0.144, and 0.23 cm^2^ for CPE, Cu(OH)_2_–Cu_2_O/PPy/CPE, and Cu(OH)_2_–Cu_2_O/PPy/F-MWCNTs/CPE, respectively. This indicates that the electroactive surface area of Cu(OH)_2_–Cu_2_O/PPy/F-MWCNTs/CPE is superior compared to the electroactive surface area of other tested electrodes.

In order to investigate the electrical interfacial properties of bare and modified electrodes, the electrochemical impedance spectroscopy (EIS) was conducted in the presence of [Fe(CN)_6_]^3/4-^ at a concentration of 10 mM containing 0.5 M KCl. Figure 4B shows the Nyquist plots of the bare CPE, Cu(OH)_2_–Cu_2_O/PPy/CPE, and Cu(OH)_2_–Cu_2_O/PPy/F-MWCNTs/CPE obtained in the frequency range from 100 KHz to 10 mHz. The EIS measurements were performed at a potential of 0.25 V vs Ag/AgCl for [Fe(CN)_6_]^3-/4-^ solution with an AC perturbation potential of 10 mV.

The charge transfer resistance (R_ct_) and the capacity of the film (C_f_) were determined by fitting impedance data using the Randles equivalent circuit. Table 2 summarizes the results of the simulation. The Nyquist plot of the bare CPE displays a large semicircle in the higher frequency, which is characteristic of the highest charge transfer resistance (282.50 Ω) and the smallest capacity of the film. The straight line in low frequency is due to the diffusion limiting process. However, after the modification of the electrode by the PPy film and copper oxide nanoparticles, the diameter of the semicircle was significantly decreased, indicating an improvement of electron transfer at the modified electrode with a smallest R_ct_ value of 24.58 Ω and high C_f_ value of 0.64 μF compared to those at the CPE. Owing to their complementary properties, PPy and Cu(OH)_2_–Cu_2_O nanoparticles play an important role in the enhancement of conductivity of the surface. After the introduction of F-MWCNTs on Cu(OH)_2_–Cu_2_O/PPy/CPE, the charge transfer resistance continued to diminish reaching a value of 2.513 Ω. This result can be attributed to the good conductivity and the high surface area of F-MWCNTs as suggested by other authors (Fard et al., 2017)**.** We can conclude that the electropolymerization of PPy on the F-MWCNT/CPE surface can generate a synergistic effect and present enhanced electrical properties. The incorporation of Cu(OH)_2_–Cu_2_O on PPy/F-MWCNTs offers a large number of active sites, and the high conductivity of Cu(OH)_2_ might further accelerate the electron transfer, despite the low conductivity of Cu_2_O (Pawar et al., 2017; Halim et al., 2021).

**TABLE 2:** Impedance parameters obtained by fitting the impedance data.

Electrode	Rs (Ω)	R_ct_ (Ω)	C_f_ (μF)
Bare CPE	**34.60**	**307.1**	**0.36**
Cu(OH)_2_–Cu_2_O/PPy/CPE	**28.58**	**28.35**	**0.64**
Cu(OH)_2_–Cu_2_O/PPy/F-MWCNTs/CPE	**30.25**	**2.51**	**0.93**

### Electrocatalytic Oxidation of Ethanol Using Modified Electrocatalysts

The catalytic performances of the modified electrocatalysts toward the ethanol oxidation reaction were evaluated using cyclic voltammetry (CV), electrochemical impedance spectroscopy (EIS), and chronoamperometry (CA) in a solution containing 0.2 M of ethanol and 0.1 M NaOH. Figure 5A shows the cyclic voltammograms recorded at different electrodes.

As expected, no response was obtained at bare CPE; however, after the deposition of copper oxide nanoparticles on the PPy/CPE surface, a well-defined anodic peak of ethanol was observed at 0.8 V vs Ag/AgCl with a current density of 4.27 mA cm^−2^ corresponding to the oxidation of ethanol molecules. After the introduction of F-MWCNTs, the current density increased 1.6-fold and reached a value of 7 mA cm^−2^ compared to that at Cu(OH)_2_–Cu_2_O/PPy/CPE at a potential of 0.85 V vs Ag/AgCl. This increase can be attributed to the good synergistic effect between PPy/F-MWCNT support and Cu(OH)_2_–Cu_2_O nanoparticles. The modified electrode presented high adsorption capability of ethanol probably due to the high surface area offered by F-MWCNTs and to the presence of a large number of amine groups of the polymer which ensures a good dispersion of Cu(OH)_2_–Cu_2_O on the electrode surface. A similar behavior was reported by Halim et al. (2021) in the oxidation of methanol. Moreover, the nanodendritic morphology of Cu(OH)_2_–Cu_2_O ensures the existence of more catalytic sites, and easy migration of species by promoting the charge transfer, leading to the improvement of the catalytic response of ethanol oxidation as suggested by other authors (Parreira et al., 2017; Zhang et al., 2018; Farsadrooh et al., 2019).

In alkaline media, the Cu(OH)_2_ layer is converted to CuOOH by the entry of OH species according to Eq. 4 (Pawar et al., 2017; Halim et al., 2021). Thereafter, ethanol is oxidized on the active CuOOH layer and forms Cu(OH)_2_ and CO_2_ (Eq. 5), which causes a high increase in the current density.

Based on the results obtained in our previous study (El Attar et al., 2021), the following mechanism could therefore be suggested for EOR at Cu(OH)_2_–Cu_2_O/PPy/F-MWCNT/CPE:

$$Cu{\left(OH\right)}_{2}+OH^{-}\to \;CuOOH+H2O+e^{-}$$ (4)

$$CuOOH+C_{2}H_{5}OH+11OH^{-}\to Cu{\left(OH\right)}_{2}+2CO_{2}+8H_{2}O+11e^{-}$$ (5)

In order to understand the electrochemical process at the interface electrode solution during the electrooxidation of ethanol, the prepared electrodes (bare CPE, Cu_2_O/PPy/CPE, and Cu_2_O/PPy/MWCNT/CPE) were examined by electrochemical impedance spectroscopy in 0.1 M NaOH in the presence of 0.2 M ethanol at a potential of 0.6 V vs Ag/AgCl in the frequency range between 100 kHz and 0.01 Hz. Figure 5B shows the impedance spectra recorded at different electrodes.

The equivalent circuit compatible with the results is presented in the inset of Figure 5B. In this circuit, R1, Q1, and R2 represent the solution resistance (Rs), a constant phase element (CPE) corresponding to the double layer capacitance, and the charge transfer resistance (R_ct_) associated with the oxidation of ethanol (Ehsani et al., 2014; Ehsani et al., 2015).

Ehsani et al. reported that the replacement of capacitor C with a constant phase element (CPE) in the equivalent circuit can lead to obtaining a satisfactory impedance simulation of ethanol electrooxidation (Ehsani et al., 2012). The most widely accepted explanation for the presence of CPE behavior is microscopic roughness of the electrocatalyst surface, causing an inhomogeneous distribution in the solution resistance as well as in the double layer capacitance. The parallel combination of charge transfer resistance R_ct_ and constant phase element CPE accounts for the injection of electrons from the conductive polymer to the back metallic contact.

The EIS results are summarized in Table 3.

**TABLE 3:** Impedance components for various electrocatalysts by fitting the experimental impedance data based on the equivalent circuit at 0.6 V vs Ag/AgCl.

Electrocatalyst	Rs (Ω)	R_ct_ (Ω)	C (μF)
Bare CPE	85.21	28000	17.61
Cu(OH)_2_–Cu_2_O/PPy/CPE	70.33	948.4	44.34
Cu(OH)_2_–Cu_2_O/PPy/F-MWCNTs/CPE	76.64	728.2	96.87

It can be seen that Cu(OH)_2_–Cu_2_O/PPy/F-MWCNTs/CPE shows the smaller semicircular diameter R_ct_ (728.2 Ω) and a higher capacitance C_f_ (96.873 μF) compared to Cu(OH)_2_–Cu_2_O/PPy/CPE (R_ct_ = 948.4 Ω, C_f_ = 44.34 μF). The decrease of R_ct_ value and the increase of C_f_ after the introduction of F-MWCNTs clearly demonstrate the ease of charge transfer and the electron flux across the electrode/electrolyte interface of Cu(OH)_2_–Cu_2_O/PPy/F-MWCNTs/CPE. The same behavior was reported by Datta et al. during the oxidation of ethanol in alkaline medium using the Pt/PANI/CNT electrocatalyst (Ding et al., 2018; Maya-Cornejo et al., 2021).

In order to evaluate the stability of the electrocatalysts, the chronoamperometric experiments of modified electrocatalysts toward EOR were investigated in 0.1 M NaOH with 0.2 M ethanol solution at 0.7 V vs Ag/AgCl for 1800 s. Figure 5C shows the obtained results.

We can notice that the three electrodes present a decline in current density trend from their maximum values of current density in the first few seconds. This decrease is due to the adsorption of incomplete oxidation products on the surface of the electrocatalyst (Chemchoub et al., 2020; El Attar et al., 2020). Cu(OH)_2_–Cu_2_O/PPy/CPE and Cu(OH)_2_–Cu_2_O/PPy/F-MWCNTs/CPE showed a very small initial drop in current before reaching stability compared to CPE. This result indicates few or no significant adsorbed intermediates species on the electrocatalyst surface (Sulaiman et al., 2017; Mozafari and Parsa, 2020b). It should be noted that the current density curve obtained at the Cu(OH)_2_–Cu_2_O/PPy/F-MWCNT/CPE catalyst reached a value of 3.57 mA cm^−2^ at 1800 s, which is much higher than the value obtained by Cu(OH)_2_–Cu_2_O/PPy/CPE (1.93 mA cm^−2^). The availability of a higher number of active sites on the surface of Cu(OH)_2_–Cu_2_O/PPy/F-MWCNTs/CPE due to the presence of F-MWCNT is probably the main reason behind this behavior (Farsadrooh et al., 2019).

The comparison of the electrocatalytic performance of our electrocatalyst with that of previously reported materials based on expensive metals is shown in Table 4. It was found that Cu(OH)_2_–Cu_2_O/PPy/F-MWCNTs/CPE shows a higher value of current density compared with that in similar works.

**TABLE 4:** Comparison of the performance of Cu(OH)_2_–Cu_2_O/PPy/F-MWCNTs/CPE with that of some other modified electrocatalysts for electrooxidation of ethanol and methanol.

Electrocatalyst	Preparation mode	Preparation time	Current density	Applied solution	Stability	Ref.
Pd/PANI-MWCNTs-SnO2/Ti	Chemical and electrochemical method	3 H	64.1 mA cm^−2^	0.5 M KOH + 1 M ethanol	20 min	Mozafari and Parsa (2020a)
PdCo PNS/PPy@MWCNT	Chemical method	More 48 H	1.65 mA cm^−2^	1 M KOH + 1 M ethanol	30 min	Fard et al. (2017)
Cu(OH)_2_–Cu_2_O/PpPD/CNF/CPE	Electrochemical method	30 min	42 mA cm^−2^	0.1 M NaOH + 1 M methanol	6 H	Halim et al. (2021)
Ni/PPy/rGO	Chemical method	–	32.94 mA cm^−2^	1 M KOH + 1 M methanol	10 min	Sarkar et al. (2019)
Cu(OH)_2_–Cu_2_O/PPy/F-MWCNTs/CPE	Electrochemical method	30 min	7 mA cm^−2^	0.1 M NaOH + 0.2 M ethanol	30 min	This work

## Conclusion

In summary, we report a new, inexpensive, and highly efficient electrocatalyst based on Cu(OH)_2_–Cu_2_O supported on PPy/F-MWCNTs for ethanol oxidation, prepared by a simple and rapid strategy. Owing to the excellent properties of F-MWCNTs, including high surface area and high conductivity, and the enrichment of the surface by amine groups of polypyrrole, a good dispersion of copper oxide on the surface is ensured. A systematic characterization of the developed electrocatalyst was conducted using FTIR spectroscopy, SEM, XRD, elemental analysis, cyclic voltammetry, and electrochemical impedance spectroscopy (EIS). The EIS of electrocatalysts in ethanol solution revealed that Cu(OH)_2_–Cu_2_O/PPy/F-MWCNTs/CPE presents a smallest R_ct_ and the highest C, which indicate a higher electronic transfer during the ethanol oxidation reaction. The Cu(OH)_2_–Cu_2_O/PPy/F-MWCNT/CPE electrocatalyst exhibited a higher electrocatalytic activity (7 mA cm^−2^) toward EOR in alkaline medium compared to Cu(OH)_2_–Cu_2_O/PPy/CPE (4.27 mA cm^−2^). It has been proved that the introduction of F-MWCNTs facilitates ethanol oxidation on the electrocatalyst surface and enhances the performance of the catalyst in terms of stability and durability confirming the collective contribution and synergistic interaction between all the material components. The good stability of electrocatalyst as well as its low cost makes this catalyst a promising candidate for direct alcohol fuel cell and other applications.

## Data Availability

The original contributions presented in the study are included in the article/Supplementary Materials, and further inquiries can be directed to the corresponding author.
