# Supplementary material for: Designing New Material Based on Functionalized Multi-Walled Carbon Nanotubes and Cu(OH)2–Cu2O/Polypyrrole Catalyst for Ethanol Oxidation in Alkaline Medium
Source: Front Chem. 2022 Feb 4;9:805654. doi: 10.3389/fchem.2021.805654 (PMC8854777; doi:10.3389/fchem.2021.805654)
Supplement: Supplementary file 1 [file DataSheet1.docx]

**Designing new material based on functionalized multi-walled carbon nanotubes and Cu(OH)_2_-Cu_2_O/Polypyrrole catalyst for ethanol oxidation in alkaline medium**

Anas EL ATTAR, Sanaa CHEMCHOUB, Mamadou Diallo KALAN, Larbi OULARBI, Mama El RHAZI*

Laboratory of Materials. Membranes, and Environment. Faculty of Science and Technology. University Hassan II of Casablanca. Morocco

*mama.elrhazi@fstm.ac.ma

**Corresponding author:**

Mama El Rhazi (Faculty of Sciences and Technologies -BP 146 Mohammedia 20650, University Hassan II of Casablanca, Morocco)

[*****elrhazim@hotmail.com](mailto:*%20elrhazim@hotmail.com)

Tel: 212 523315352 Fax: 212 523315353

**Figure S1A.** FTIR analysis of MWCNTs before and after the acid treatment.

~~
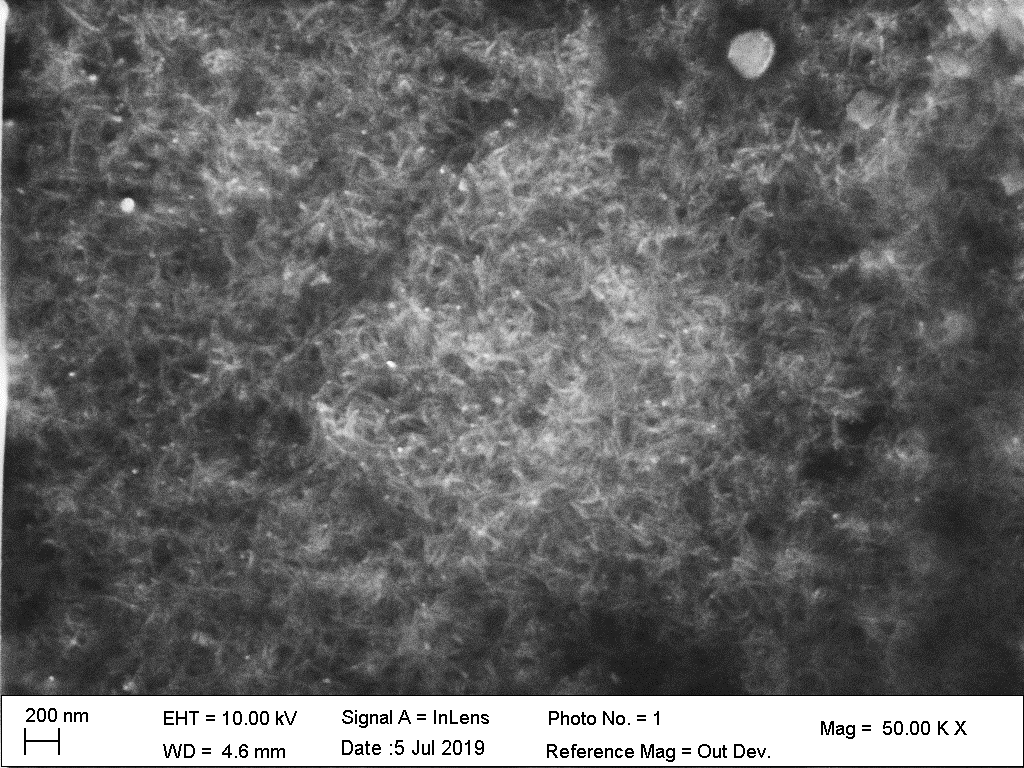
~~

**Figure S1B.** SEM images of MWCNTs after the acid treatment.

**Figure S2.** Typical cyclic voltammograms of: PPy/CPE and PPy/F-MWCNTs/CPE in 0.5 M LiClO_4_ at a scan rate of 50 mV s^−1^
